# Associations between change in physical activity and sedentary time and health-related quality of life in older english adults: the EPIC-Norfolk cohort study

**DOI:** 10.1186/s12955-023-02137-7

**Published:** 2023-06-22

**Authors:** Dharani Yerrakalva, Samantha Hajna, Marc Suhrcke, Katrien Wijndaele, Kate Westgate, Kay-Tee Khaw, Nick Wareham, Soren Brage, Simon Griffin

**Affiliations:** 1 Department of Public Health and Primary Care, University of Cambridge School of Clinical Medicine, Cambridge, UK; 2 MRC Epidemiology Unit, University of Cambridge, School of Clinical Medicine, Cambridge, UK; 3 University of York, York, UK

**Keywords:** Older adults, Physical activity, Sedentary, Quality of life

## Abstract

**Background:**

No previous studies have examined the associations between changes in objectively-measured physical behaviours with follow-up QoL in older adults. Based on cross-sectional evidence, it is biologically plausible that such associations exist. If so, this bolsters the case for the commissioning of activity interventions and for including QoL as an outcome in trials of such interventions.

**Methods:**

We assessed physical behaviours (total physical activity, moderate-to-vigorous physical activity (MVPA), light physical activity, total sedentary time and prolonged sedentary bout time) for 7 days using hip-worn accelerometers at baseline (2006–2011) and follow-up (2012–2016) and health-related quality-of-life (QoL) using EQ-5D questionnaires at follow-up in 1433 participants (≥ 60 years) of the EPIC (European Prospective Investigation into Cancer)-Norfolk study. The EQ-5D summary score was used, with 0 as the worst to 1 as best perceived quality-of-life. We evaluated the prospective associations of baseline physical behaviours with follow-up QoL, and of changes in behaviours with follow-up QoL using multi-level regression.

**Results:**

On average, MVPA decreased by 4.0 min/day/year (SD 8.3) for men and 4.0 min/day/year for women (SD 12.0) between baseline and follow-up. Total sedentary time increased by an average 5.5 min/day/yr (SD 16.0) for men and 6.4 min/day/yr (SD 15.0) for women between baseline and follow-up. Mean (SD) follow-up time was 5.8 (1.8) years.

We found that higher baseline MVPA and lower sedentary time was associated with higher subsequent QoL (e.g. 1 h/day greater baseline MVPA was associated with 0.02 higher EQ-5D score, 95% CI 0.06, 0.36). More pronounced declines in activity were associated with worse Hr-QoL (0.005 (95% CI 0.003, 0.008) lower EQ-5D per min/day/yr decrease in MVPA). Increases in sedentary behaviours were also associated with poorer QoL (0.002 lower EQ-5D, 95% CI -0.003, -0.0007 per hour/day/yr increase in total sedentary time).

**Conclusions:**

Promotion of physical activity and limiting sedentary time among older adults may improve quality-of-life, and therefore this relationship ought to be included in future cost effectiveness analyses so that greater commissioning of activity interventions can be considered.

**Supplementary Information:**

The online version contains supplementary material available at 10.1186/s12955-023-02137-7.

## Introduction

More physical activity and less sedentary time are associated with reduced risk of morbidity such as diabetes, depression, cardiac disease and cancer, and premature mortality [1–7]. However, a significant proportion of UK older adults do not meet current physical activity guidelines [8]. The most recent UK guidelines state older adults should aim to accumulate 150 min of moderate intensity aerobic activity per week [9]. Additionally, they state older adults should break up prolonged periods of being sedentary with light activity when physically possible, or at least with standing, as this has distinct health benefits for older people.

Interventions to prevent declines in physical activity and increases in sedentary time have not achieved sustained changes in behaviour beyond 12 months [10–13]. Health-related quality of life (QoL) is a comprehensive measure of health and wellbeing, which can be used to assess healthy ageing, complementing standard measures of mortality and morbidity [14]. Lower QoL is inversely associated with risk of hospitalisation [15], adverse post-hospitalisation outcomes [16], and premature mortality [17]. It is also used to inform decisions about the commissioning of health care. Effects of interventions to improve QoL in older adults have varied [18].

Understanding the relationship of physical activity and sedentary time with QoL enables assessment of whether and how changes in activity might translate into improvements in QoL. In future, this could inform interpretation of cost-effectiveness analyses underpinning resource allocation decisions. Previous assessments of the cost-effectiveness of interventions to promote activity may have underestimated their value, as effects on QoL are commonly not taken into account. Instead the focus has traditionally been on mortality and disease incidence outcomes [19]. If there is a strong and causal relationship between physical activity and QoL, this could strengthen the case for investment and commissioning of interventions to promote activity. Further, given that QoL may be more important to older adults than risk of morbidity and premature mortality, such research could be incorporated into motivational messaging in activity-based interventions.

It is biologically plausible that better physical behaviour profiles are causally associated with subsequent Hr-QoL. For example, higher physical activity levels are associated with better physical function/mobility [20, 21], ability to do self-care [22] and other usual activities [23], reduced levels of pain [24] and anxiety/depression [25]. These are all domains of Hr-QoL, and could be potential mechanisms through which physical behaviours may effect Hr-QoL. Higher levels of physical activity and less time spent in sedentary behaviours leads to lower risk of many chronic conditions. These conditions can themselves lead to deterioration in components of Hr-QoL. For example, high levels of physical activity are associated with a lower risk of arthritis (a cause of pain, a component of Hr-QoL) and cognitive decline (a cause of reduced ability to self-care) [26, 27]. Reduced sedentary time and increased physical activity are also linked to improved social functioning, reduced loneliness and social isolation [28] which could all promote better Hr-QoL.

Our current understanding of these relationships is limited in four important ways. Firstly, studies have been almost exclusively cross-sectional limiting interpretation of causality. Secondly, the few existing longitudinal studies have all used subjective rather than more precise and less biased objectively-assessed measures of activity [29–35]. There have been no longitudinal studies of associations between QoL and objectively-measured activity in adults. Examining longitudinal associations allows us to examine whether there is a directional nature to any observed association, and therefore gives added insight in comparison to cross-sectional analysis. Thirdly, older adults have been neglected in studies of these associations from our review of the literature [36–38].

Finally, there are no studies examining the prospective relationship between prolonged sedentary bouts and QoL. Prolonged sedentary bouts are thought to be particularly detrimental to health independent of total time spent sedentary [31–35] though the mechanism of this remains unclear. Prolonged sedentary bouts are associated with worse metabolic health outcomes such as metabolic syndrome [36–38] and poor glycaemic control [31, 39]. Therefore, less time in prolonged sedentary bouts could theoretically lead to better QoL through fewer chronic diseases. The advent of objective measures of sedentary time has allowed measurement of sedentary patterns such as prolonged sedentary bouts. Though traditionally researchers have investigated the risk factor of total daily sedentary time, more recently there has been interest in looking at time spent in prolonged bouts (e.g. time in bouts of more than 30 min) as it may be easier to get individuals to break up prolonged bouts than reduce total time (e.g. frailer older adults who are unable to participate in physical activity or stand for prolonged periods).

There is a need for large longitudinal studies that utilise objectively-assessed measures of physical activity and sedentary time in this population. We aimed to describe the prospective associations between accelerometer-assessed activities (total physical activity, total sedentary time, prolonged sedentary bout time, light physical activity (LPA), moderate-to-vigorous intensity PA (MVPA) and QoL in a large sample of older adults.

## Methods

We used data from the EPIC-Norfolk (European Prospective Investigation into Cancer-Norfolk) study, a prospective cohort of over 25,500 adults living in the UK [40] who participated in up to five health-checks. Participants were similar to a national population sample in terms of anthropometry, serum lipids, and blood pressure (Health Survey for England) [41]. We used data from two assessment time-points, hereafter described as baseline (2006–2011) and follow-up (2012–2016). Physical behaviour was assessed by accelerometer at baseline (*n* = 3,784) and follow-up (*n* = 4,788). QoL was measured by questionnaire at follow-up (*n* = 2,113). We restricted our analyses to participants who were aged ≥ 60 years at baseline and who had valid accelerometry and QoL data at relevant assessments.

### Accelerometry

Estimates of physical behaviours were collected via hip-mounted accelerometers at baseline and follow-up. At baseline, participants wore uniaxial accelerometers (Actigraph GT1M™, USA). At follow-up, participants wore triaxial accelerometers (GT3X™, Actigraph, USA). Participants were asked to wear accelerometers on their right hip for seven days except when bathing, swimming or sleeping. Harmonisation of the data from the two accelerometers was completed using previously described methods [42, 43] and activity was integrated into 60-s epochs before summation [44, 45]. Non-wear time was defined as continuous zero counts of ≥ 90 min [46]. In order to deal with overnight wear, we overlaid self-report sleep timings at epoch level for days with wear-time > 19 h and excluded data accordingly. Variables derived from accelerometry data were total physical activity, MVPA, LPA, total sedentary time, and prolonged sedentary bout time (bouts ≥ 30 min). Total physical activity was calculated by total activity counts divided by wear time (counts/minute). The cut-offs used to define intensity-related behaviours were < 100 counts per minute (cpm) for sedentary time, 100–808 cpm for LPA, and ≥ 809 cpm for MVPA [42, 46–50], in units minutes/day. We calculated the rate of change of accelerometer-assessed variables (min/day/year) as the difference between values at baseline and follow-up divided by the time between assessments. Participants with ≥ 4 days of valid wear-time (≥ 10 h of wear time each day) for each assessment were included in this analysis.

### QoL

QoL was measured in follow-up using the EQ-5D-3L, a validated self-completion questionnaire [51] which was mailed to participants. The EQ-5D descriptive system includes domains of mobility, self-care, usual activities, pain and anxiety/depression. It can be summarised using a single value, which reflects how good or bad a person’s QoL is according to the preferences of the general population of a country [52, 53], with 0 as the worst to 1 as best perceived QoL. We derived the summary value using previously described methods utilising the UK value set cited in the EQ5D3L guide.

### Covariates

Baseline sociodemographic factors were age, sex, smoking status (never, former, current), body mass index (BMI), and occupation (Registrar-General's Social Classification). We also utilised job status (job vs no job), educational status (O level or lower vs A level or higher), chronic disease status (history of either myocardial infarction, stroke, cancer or diabetes mellitus), marital status (single, married, widowed, separated, divorced) and household financial circumstances (“in general, do you or your family have more money than you needed, just enough or not enough?”). All these were assessed via self-completed questionnaire. BMI (kg/m^2^) was calculated based on weight and height, which were measured by trained staff following standard operating procedures.

### Statistical analyses

We calculated descriptive statistics for all socio-demographic, activity and QoL measures of interest and examined differences between participants included in the main analyses and those that we excluded. We also calculated descriptive statistics for annual change in physical behaviour measures.

We undertook longitudinal analyses using multivariable linear regression to estimate *firstly*, the association between baseline physical behaviours and follow-up QoL, and *secondly,* the association between change in behaviours (baseline to follow-up) and follow-up QoL.

We examined each of these associations using three models. We accounted for factors such as age and sex, which are likely confounders, by fitting them into model 2. Older age and being female have been associated with lower Hr-QoL, lower MVPA and higher sedentary time [54, 55]. Other biologically plausible confounders identified a priori were added into model 3. Model 1 was adjusted for season at baseline and follow-up [56, 57], time difference between baseline and follow-up, and accelerometer wear time. Model 2 was additionally adjusted for age and sex. Model 3 was the same as Model 2 but with additional adjustment for baseline job status, smoking status, BMI, chronic disease status, occupational class, marital status, education level and household financial circumstances. For the analyses of change in physical behaviour, adjustment was also made for baseline behaviour across all models. In order to further contextualise our results, we performed a regression of QoL against age, with adjustment for sex, to estimate QoL decline per chronological year of age.

In sensitivity analyses, we examined if valid day inclusion criteria (≥ 5 vs ≥ 4 days of valid data) and behaviour intensity cut-points (i.e. using 809 cpm vs 2,020 cpm to delineate LPA and MVPA) influenced our results. All analyses were conducted in STATA 15.0 (StataCorp, TX, USA) using complete case analyses.

## Results

There were 1584 participants adults aged ≥ 60 years that had QoL and activity measurements at appropriate assessments making them eligible for inclusion. Of these, 10 individuals at baseline and 15 individuals at follow-up were excluded due to having < 4 valid days of accelerometry data. A further 126 participants were excluded due to missing covariates, leaving a total of 1433 participants (90%). Participants had an average (SD) age of 70 [7] years at baseline and 54.7% were women (Table 1). Included participants were socio-demographically similar to those excluded (Supplementary Table 1).

**Table 1:** Characteristics of included participants from the EPIC-Norfolk study 2006–2016 with physical behaviours at baseline and follow-up and quality of life measurement at follow-up (*n* = 1584)

Characteristics		*Frequency*	Percent (%)
**Sex**	Male	645	45.3
	Female	788	54.7
**Age**	< 65y	557	38.9
	65-70y	367	25.6
	> 70y	509	35.5
**Ethnicity**	White	1430	99.8
	Other	3	0.2
**Occupational Classification**	Professional	133	9.3
	Manager	598	41.7
	Skilled non-manual	228	15.9
	Skilled manual	292	20.4
	Semi-skilled	156	10.9
	Non-skilled	26	1.8
**Employed**	No	1151	80.3
	Yes	282	19.7
**Further Education level**	O-level or lower	631	44.0
	A-level or higher	802	56.0
**Smoking Status**	Current	44	3.1
	Former	671	46.8
	Never	718	50.1
**History of Chronic Disease**	No	1161	81.0
	Yes	27	19.0
**Body Mass Index (kg/m**^**2**^**)**	< 25	504	35.2
	25- < 30	671	46.8
	30- < 35	215	15.0
	≥ 35	43	3.0

This table shows the percentage spread across categories of demographic and clinical characteristics for those included (*n* = 1433). Further education level categories include O level or lower (UK national qualification to age 16) vs A level or higher (UK national qualification over age 16). Baseline characteristics were measured 2006–2011

Mean (SD) time between baseline and follow-up was 5.8 (1.8) years. On average, MVPA decreased by 4.0 min/day/year (SD 8.3) for men and 4.0 min/day/year for women (SD 12.0) between baseline and follow-up (Table 2). LPA decreased by 4.0 min/day (SD11) and 3.5 min/day for women (SD 8.8). Total sedentary time increased by an average 5.5 min/day/yr (SD 16.0) for men and 6.4 min/day/yr (SD 15.0) for women between baseline and follow-up. Prolonged sedentary bout time increased by 9.3 min/day (SD 19.8) for men and 9.0 min/day for women (SD 16.8). After adjustment for sex, participants had a 0.0069 lower EQ-5D score per year of older age (95% CI -0.0083, -0.0054).

**Table 2:** Physical behaviours at baseline and follow-up and QoL at follow-up of participants in the EPIC-Norfolk study 2006–2016 (*n* = 1584)

	**Mean (SD) at baseline**		**Mean (SD) at follow-up**		**Mean Annual Change (SD)**	
	**Men**	**Women**	**Men**	**Women**	**Men**	**Women**
**Total PA (cpm)**	252 (126)	233 (130)	220 (104)	233 (130)	-9.2 (22.1)	-8.9 (20)
**Total sedentary time (min/day)**	586 (84)	542 (81)	600 (83)	568 (79)	5.5 (16)	6.4 (14.7)
**Prolonged sedentary bout (min/day)**	228 (101)	178 (86)	259 (108)	215 (97)	9.3 (19.8)	9.0 (16.8)
**LPA **^**100–809 cpm**^** (min/day)**	207 (54)	238 (54)	194 (55)	224 (56)	-4.0 (11)	-3.5 (8.8)
**LPA **^**100–2020 cpm**^** (min/day)**	264 (78)	297 (76)	243 (79)	270 (78)	-6.8 (14.6)	-6.8 (15.8)
**MVPA **^**809 cpm**^** (min/day)**	78 (48)	76 (45)	68 (43)	66 (42)	-4.0 (8.3)	-4.0 (12)
**MVPA **^**2020 cpm**^** (min/day)**	23.0 (20.7)	18.3 (16.6)	21.4 (21.3)	16.5 (16.4)	-1.0 (4.5)	-0.8 (3.6)
**EQ-5D**	-	-	0.9 (0.2)	0.9 (0.2)	-	-

This table shows the mean values of activity measures and Hr-QoL measures at baseline (2006–2011) and follow-up (2012–2016). *TPA* total physical activity, *MVPA*   moderate-to-vigorous activity, *LPA* light physical activity, *SD* standard deviation. A dash(-) denotes that the measure was not performed at that time point

### Association of baseline activity with follow-up QoL

**Fig. 1:**
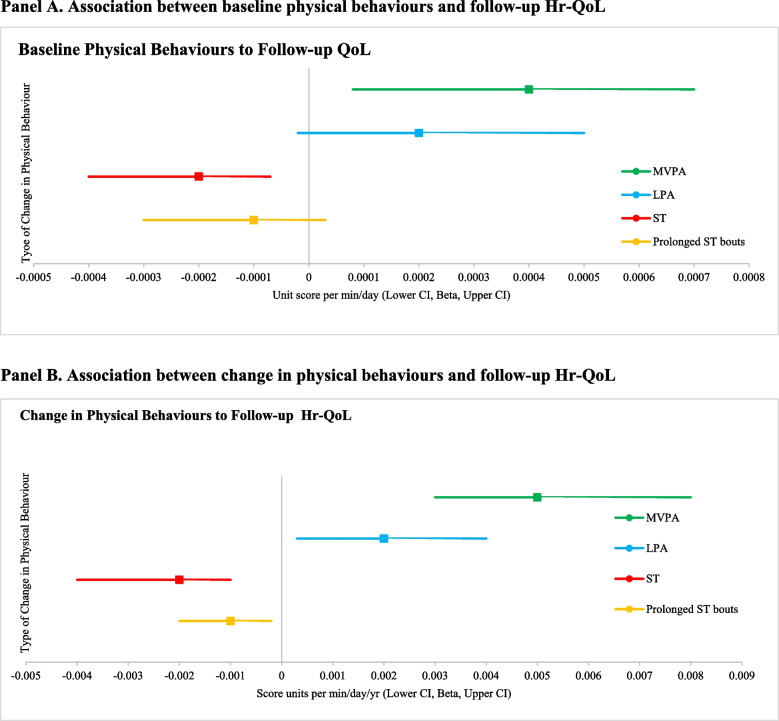
Association between physical behaviours at and quality of life for included participants from the EPIC-Norfolk study 2006–2016 (*n* = 1584). For all panels, MVPA is in green, LPA is in blue, ST is in red and Prolonged ST bouts is in orange. Beta is indicated by central square, 95% CI is indicated by the line. Baseline measures were performed 2006–2011 and follow-up measures were done 2012–2016. In Panel A, results are from model 3 which are adjusted for season and wear time at baseline, age, sex, job status, smoking status, occupational class, retirement status, BMI, ethnicity, chronic disease status, marital status and household financial status. In Panel B, results are additionally adjusted for season and accelerometer wear-time at baseline and follow-up, and baseline activity. **A** Association between baseline physical behaviours and follow-up Hr-QoL. **B** Association between change in physical behaviours and follow-up Hr-QoL

Higher baseline total physical activity and MVPA were associated with higher subsequent QoL (Fig. 1, Supplementary Table 2). Specifically, a 100 cpm/day higher total physical activity and an hour/day higher MVPA were associated with a 0.02 unit (95% CI 0.005, 0.03) and a 0.02 unit (95% CI 0.06, 0.36) higher EQ-5D score, respectively. Higher total sedentary time was associated with lower subsequent QoL (1 h/day higher sedentary time was associated with 0.01 unit higher EQ5D score, 95% CI -0.02, -0.004). However, LPA and prolonged sedentary bouts were not statistically significantly associated with Hr-QoL.

### Association of change in activity with follow/up QoL

Smaller declines in all physical activity measures were associated with better QoL at follow-up. Greater increases in all sedentary variables were associated with poorer QoL (Supplementary Table 3). Every hour/day/yr increase in MVPA was associated with 0.3 higher EQ-5D units (95% CI 0.2, 0.5). Every hour/day/yr increase in LPA was associated with 0.12 higher EQ-5D units (95% CI 0.02, 0.2). Every hour/day/year increase in total sedentary time was associated with 0.12 fewer subsequent EQ-5D units (95% CI -0.2, –0.06). Every hour/day/year increase in prolonged sedentary bout time was associated with 0.06 fewer subsequent EQ-5D units, 95% CI -0.1, -0.01).

### Sensitivity analyses

The results using different cut-points for MVPA and LPA showed similar results (Supplementary Tables 2–3). There were no important differences between our main results (i.e. utilising ≥ 4 days of valid wear-time) and results using stricter inclusion criteria for accelerometry measures (i.e. ≥ 5 days of valid wear-time) (data not shown).

## Discussion

We found that higher baseline MVPA and lower total sedentary time were associated with higher QoL approximately 6 years later. Further, smaller declines over time in MVPA and LPA, and smaller increases in total sedentary time and prolonged sedentary bout time were associated with better QoL. Taken together, this suggests that promotion of physical activity and limiting sedentary time in individuals may be an appropriate approach to achieving a higher absolute QoL.

No previous studies have examined the associations between baseline objectively-measured physical behaviours and follow-up QoL in adults of any age. Two studies examined this association using self-reported physical activity [36, 38]. Balboa-Castillo et al. found that greater self-reported physical activity and lower sedentary time were independently associated with better subsequent QoL in older adults (70.3 ± 5.6 years, *n* = 1,097) [36]. Dugan et al. found that in women (45.9 ± 2.7 years, *n* = 2,400) higher self-reported physical activity was associated with higher QoL 3 years later [38]. Our study goes beyond this by showing that greater baseline MVPA and lower total sedentary time, as assessed using accelerometry, were associated with higher subsequent QoL.

There are also no previous studies that have examined the association between changes in objectively-measured physical behaviours with follow-up QoL. Only one study of adults examined the association between change in self-reported physical activity and subsequent QoL. Wolin et al. found that women (aged 40–67 years) who self-reported increased physical activity had higher subsequent QoL, compared to women reporting stable physical activity levels over 8 years follow-up [37]. Our study is the first to demonstrate that greater objectively-assessed declines in MVPA and LPA, and increases in all sedentary variables, were negatively associated with subsequent QoL.

To put our results in clinical context, we found that increases in sedentary time of the magnitude achieved in intervention studies (1 h/day/year) led to a 0.002 points/year lower subsequent EQ-5D score [58, 59]. We also found that change in MVPA of the magnitude (but not direction) seen in RCTs (10 min/day/year increase) led to a 0.005 points/year lower subsequent EQ5D score [59, 60]. A 0.1 point improvement in EQ-5D score has been associated with a 6.9% reduction in mortality risk and a 4.2% reduction in risk of hospitalisation [61]. This level of improvement could mitigate the age-related decline in QoL that we observed in this cohort (-0.0069 points per year of older age). Therefore our results suggest that future interventions promoting improvements in activity profiles could lead to small clinical improvements in QoL. In addition, our work suggests that promoting LPA and reducing prolonged sedentary bout time, potentially easier targets, could also lead to improvement in QoL.

### Strengths and limitations

Our work has several strengths. Firstly, we used objective measures of physical activity and sedentary time. Secondly, EPIC-Norfolk is a large population-based cohort providing greater power and ability to adjust for confounding in analyses. Thirdly, the longitudinal design of the EPIC-Norfolk study gave us the opportunity to examine prospective associations between activity and QoL, albeit reverse causality cannot be excluded. Further, we used valid measures of QoL which appear stable over time [62, 63]. In addition, we used summary scores for the measure of QoL (EQ-5D), aggregate scores of the original domains. This had the benefit of reducing multiple testing and avoiding reduction in statistical power, and examines a more global measure of QoL [64].

There are also several limitations. EPIC-Norfolk participants were slightly healthier than the general population [41] at the 1st health-check (1993–1997), and additionally those who participated at the 3^rd^ health-check (2004–2011) were even healthier (e.g. lower blood pressure and cholesterol) than those who participated in the 1^st^ health-check only [40] likely due to healthy volunteer bias and selective attrition. Though accelerometers provide objective measures in contrast to self-report, they do not collect information on the type of activity being done (e.g. upper body movements, standing still) which can lead to misclassification bias. Further, they cannot record water-based activity, which can lead to missing data. We minimised non-wear misclassification (i.e. not wearing the accelerometer versus being still) by using an algorithm with a threshold of ≥ 90 min [65].

## Conclusions

We found that higher levels of physical activity and fewer minutes spent sedentary measures were associated with better subsequent QoL in a population of UK older adults. This work therefore supports the case for promotion of physical activity and limitation of sedentary time. QoL outcomes should be included in future intervention trials and cost effectiveness analyses. Our results add to the evidence for the wider benefits of interventions promoting physical activity and highlight the need for additional effective interventions.

## Supplementary Information

**Additional file 1: Supplementary Table 1.** Characteristics of included participants versus those excluded from the EPIC-Norfolk study 2006-2016. **Supplementary Table 2.** Association of baseline physical activity and sedentary time with follow-up QOL in the EPIC-Norfolk study 2006-2016. **Supplementary Table 3.** Association of change in physical activity and sedentary time with follow-up QOL in the EPIC-Norfolk study 2006-2016

## Data Availability

Data from the EPIC-Norfolk study must be requested directly from their data request team by completing a data request form and emailing it to epic-norfolk@mrc-epid.cam.ac.uk.
