# Supplementary material for: Associations between change in physical activity and sedentary time and health-related quality of life in older english adults: the EPIC-Norfolk cohort study
Source: Health Qual Life Outcomes. 2023 Jun 22;21:60. doi: 10.1186/s12955-023-02137-7 (PMC10288723; doi:10.1186/s12955-023-02137-7)
Supplement: Supplementary file 1 — Additional file 1: Supplementary Table 1. Characteristics of included participants versus those excluded from the EPIC-Norfolk study 2006-2016. Supplementary Table 2. Association of baseline physical activity and sedentary time with follow-up QOL in the EPIC-Norfolk study 2006-2016. Supplementary Table 3. Association of change in physical activity and sedentary time with follow-up QOL in the EPIC-Norfolk study 2006-2016 [file 12955_2023_2137_MOESM1_ESM.docx]

**Supplementary Table 1: Characteristics of included participants versus those excluded from the EPIC-Norfolk study 2006-2016**

| **Characteristics** | | **Percent of Included participants (n=1433)(%)** | **Percent of Excluded participants (n=151)(%)** |
| --- | --- | --- | --- |
| **Sex** | Men | 45.3 | 42.2 |
|  | Women | 54.7 | 57.8 |
| **Ethnicity** | White | 99.8 | 99.8 |
|  | Other | 0.2 | 0.2 |
| **Occupational Classification** | Professional | 9.3 | 7.3 |
|  | Manager | 41.7 | 39.5 |
|  | Skilled non-manual | 15.9 | 17.0 |
|  | Skilled manual | 20.4 | 22.4 |
|  | Semi-skilled | 10.9 | 14.0 |
|  | Non-skilled | 1.8 | 1.8 |
| **Employed*** | No | 80.3 | 81.5 |
|  | Yes | 19.7 | 18.5 |
| **Further Education level** | O-level or lower | 44.0 | 41.9 |
|  | A-level or higher | 56.0 | 58.1 |
| **Smoking Status*** | Current | 3.1 | 5.1 |
|  | Former | 46.8 | 43.8 |
|  | Never | 50.1 | 51.1 |
| **History of Chronic Disease *** | No | 81.0 | 89.5 |
|  | Yes | 19.0 | 10.5 |
| **Body Mass Index (**kg/m^2^)* | <25 | 35.2 | 30.5 |
|  | 25-<30 | 46.8 | 47.9 |
|  | 30-<35 | 15.0 | 15.3 |
|  | ≥35 | 3.0 | 6.3 |

This table shows the percentage spread across categories of participant characteristics for those included versus excluded. Further education level categories include O level or lower (UK national qualification to age 16) vs A level or higher (UK national qualification over age 16).

**Supplementary Table 2: Association of baseline physical activity and sedentary time with follow-up QOL in the EPIC-Norfolk study 2006-2016 (n = 1584)**

|  | **Follow-up Hr-QoL (unit score of Ed5D)** | | | | | |
| --- | --- | --- | --- | --- | --- | --- |
| **Model Number** | 1 | | 2 | | 3 | |
|  | **β** | 95% CI | **β** | 95% CI | **β** | 95% CI |
| **Baseline Activity Measure** |  |  |  |  |  |  |
| Total PA (per 100 counts/min) | 0.03 | 0.02, 0.04 | 0.02 | 0.01, 0.03 | 0.02 | 0.005, 0.03 |
| MVPA (per min/day) | 0.0007 | 0.0004, 0.0009 | 0.0005 | 0.0002, 0.0007 | 0.0004 | 0.00008, 0.0007 |
| LPA (per min/day) | 0.0004 | 0.001, 0.0006 | 0.0003 | 0.00005, 0.0005 | 0.0002 | -0.00002, 0.0005 |
| Total sedentary time (per min/day) | -0.0004 | -0.0006, -0.0002 | -0.0003 | -0.0005, -0.0001 | -0.0002 | -0.0004, -0.00007 |
| Prolonged sedentary bout time (per min/day) | -0.0003 | -0.0004, -0.0002 | -0.0002 | -0.0004, -0.00006 | -0.0001 | -0.0003, 0.00003 |
| *Sensitivity Analysis (MVPA defined as ≥2020cpm, LPA defined as 100-2019 cpm)* | | | | |  |  |
| MVPA (per min/day) | 0.002 | 0.001, 0.003 | 0.001 | 0.0005, 0.002 | 0.0007 | -0.00005, 0.001 |
| LPA ( per min/day) | 0.0004 | 0.0002, 0.0006 | 0.0002 | 0.00006, 0.0004 | 0.0002 | 0.00005, 0.004 |

This table shows the associations of baseline physical activity and sedentary time with follow-up Hr-QoL, measured using EQ-5D. Season was coded using sine and cosine functions; spring = sin (2*π* day of year /365.25), winter = cos (2 *π* day of year/365.25). The main analysis used activity variable cut-points of ≥809 cutpoint for MVPA and 100-808 cutpoint for LPA. The sensitivity analyses shown here used ≥2020cpm for MVPA and 100-2019cpm for LPA. Beta coefficients are displayed here as QoL differences per min/day for MVPA, LPA and ST and prolonged sedentary bout time and per 100 counts/min for Total PA.

95% CI=95% confidence interval, TPA=total physical activity, MVPA=moderate-to-vigorous activity, LPA=light physical activity.

^a^Model 1 was adjusted for season and wear time at baseline and follow-up.

^b^Model 2 was the same as model 1 plus mutually adjusted for age and sex.

^c^Model 3 was the same as Model 2 plus mutually adjusted for potential socioeconomic and environmental confounders (job status, smoking status, occupational class, retirement status, BMI, ethnicity, chronic disease status, marital status and household financial status).

**Supplementary Table 3: Association of change in physical activity and sedentary time with follow-up QOL in the EPIC-Norfolk study 2006-2016 (n = 1584)**

|  | **Follow-up QoL (unit score of Ed5D)** | | | | | |
| --- | --- | --- | --- | --- | --- | --- |
| **Model Number** | 1 | | 2 | | 3 | |
|  | **β** | **95%CI** | **β** | **95%CI** | **β** | **95%CI** |
| **Change in Activity Measure** |  |  |  |  |  |  |
| TPA(per 100 cpm/yr) | 0.3 | 0.2, 0.3 | 0.2 | 0.1, 0.3 | 0.002 | 0.0009, 0.003 |
| MVPA (per min/day/yr) | 0.007 | 0.004, 0.009 | 0.005 | 0.003, 0.008 | 0.005 | 0.003, 0.008 |
| LPA (per min/day/yr) | 0.003 | 0.001, 0.005 | 0.003 | 0.0008, 0.005 | 0.002 | 0.0003, 0.004 |
| Total sedentary time (per min/day/yr) | -0.003 | -0.005, -0.002 | -0.003 | -0.004, -0.002 | -0.002 | -0.004, -0.001 |
| Prolonged ST bout time (per min/day/yr) | -0.002 | -0.003, -0.001 | -0.002 | -0.003, -0.0007 | -0.001 | -0.002, -0.0002 |
| ***Sensitivity Analysis (MVPA defined as ≥2020cpm, LPA defined as 100-2019 cpm)*** | | | | | | |
| MVPA (per min/day/yr) | 0.01 | 0.007, 0.02 | 0.01 | 0.004, 0.02 | 0.008 | 0.003, 0.01 |
| LPA (per min/day/yr) | 0.003 | 0.002, 0.005 | 0.003 | 0.001, 0.004 | 0.002 | 0.0005, 0.003 |

This table shows the associations of change in physical activity and sedentary time with follow-up Hr-QoL, measured by EQ-5D **(n=655)**. Coding of season was using sine and cosine functions; spring = sin (2*π* day of year /365.25), winter = cos (2 *π* day of year/365.25)]. The main analysis used activity variable cut-points of ≥809 cutpoint for MVPA and 100-808 cutpoint for LPA. The sensitivity analyses shown here used ≥2020cpm for MVPA and 100-2019cpm for LPA.

TPA=total physical activity, MVPA=moderate-to-vigorous activity, LPA=light physical activity.

^a^Model 1 was adjusted for season and wear time at baseline and follow-up, baseline activity measure and QoL measure.

^b^Model 2 was the same as model 1 plus mutually adjusted for age and sex.

^c^Model 3 was the same as Model 2 plus mutually adjusted for potential socioeconomic and environmental confounders (job status, smoking status, occupational class, retirement status, BMI, ethnicity, chronic disease status, marital status and household financial status).
